# Death of Dr. Keith, of Aberdeen

**Published:** 1871-04

**Authors:** 


Death of Dr. Keith, of Aberdeen.—This distinguished surgeon died at Edinburgh on the 5th ult. His experience in lithotomy was perhaps larger than that of any of his contemporaries, and his success in that operation was remarkable. A most distinguished operating surgeon and an able teacher, he was above the petty prejudices which too often mar the fame of otherwise good and noble men, and was one of the first to acknowledge the good to be derived from acupressure, though that admirable mode of arresting hæmorrhage was not derived from strictly surgical parentage. In conjunction with Dr. Pirie he published an important work on this subject, which may yet serve to give this simple and effective procedure its true position in surgery. Dr. Keith was greatly loved as a man as well as a surgeon, and few will be more missed among his contemporaries of the granite city.—*Edinburg Med. Jour.*

